# Supplementary material for: Comparing the impact of systemic pituitary adenylate-cyclase-activating polypeptide (PACAP) and calcitonin gene-related peptide (CGRP) on motion-induced nausea and balance behaviors in mice
Source: PLoS One. 2025 Nov 18;20(11):e0334444. doi: 10.1371/journal.pone.0334444 (PMC12626257; doi:10.1371/journal.pone.0334444)
Supplement: S1 Table — Recovery period is calculated by taking the x-intercept (minutes) and subtracting 20 minutes (duration of provocative rotation from t = 0 to t = 20 mins). (DOCX) [file pone.0334444.s001.docx]

| **Analyses from X-Intercept Quadratic Model across sex and treatment for Head Temperature Profiles** | | | | | | |
| --- | --- | --- | --- | --- | --- | --- |
|  |  |  |  |  |  |  |
| **Variables** | **Vehicle (F)** | **Vehicle (M)** | **PACAP-38 (F)** | **PACAP-38 (M)** | **CGRP (F)** | **CGRP (M)** |
|  | **Best-fit values** | | | | | |
| ***slope1*** | 0.096 | 0.090 | 0.039 | 0.065 | 0.062 | 0.116 |
| ***slope2*** | 0.002 | 0.002 | 0.0008 | 0.001 | 0.001 | 0.003 |
| ***X-intercept (mins)*** | 39.21 | 40.36 | 47.97 | 46.67 | 46.95 | 41 |
| ***Recovery period (mins)*** | 19.21 | 20.36 | 27.97 | 26.67 | 26.95 | 21 |
|  | **95% CI (profile likelihood)** | | | | | |
| ***slope1*** | 0.08 to 0.11 | 0.07 to 0.11 | 0.02 to 0.06 | 0.029 to 0.09 | 0.04 to 0.08 | 0.09 to 0.14 |
| ***slope2*** | 0.002 to 0.003 | 0.001 to 0.003 | 0.0002 to 0.002 | 0.0003 to 0.002 | 0.0007 to 0.002 | 0.002 to 0.004 |
| ***X-intercept*** | 37.22 to 42.02 | 37.95 to 44.08 | 39.80 to 110.2 | 40.06 to 78.44 | 41.46 to 61.39 | 38.33 to 45.29 |
|  | **Goodness of Fit** | | | | | |
| ***Degrees of Freedom*** | 18 | 18 | 18 | 18 | 18 | 18 |
| ***R squared*** | 0.768 | 0.697 | 0.470 | 0.344 | 0.615 | 0.702 |
| ***Sum of Squares*** | 0.65 | 0.72 | 0.78 | 1.54 | 0.76 | 1.33 |
| ***Sy.x*** | 0.19 | 0.2 | 0.2 | 0.29 | 0.21 | 0.27 |

**S1. Table 1**. To evaluate recovery from hypothermia, an x-intercept quadratic model was used, with a constraint imposed to detect x-intercepts after time t = 20 minutes (when provocative motion is turned off). Recovery period is calculated by taking the x-intercept (minutes) and subtracting 20 minutes (duration of provocative rotation from t = 0 to t = 20 mins).
